# Type I interferon signaling in microglia drives synaptic engulfment and neuronal loss following traumatic brain injury

**DOI:** 10.21203/rs.3.rs-9785030/v1

**Published:** 2026-06-06

**Authors:** Brittany P. Todd, Zili Luo, Molly J. E. Larson, Polly J. Ferguson, Alexander G. Bassuk, Elizabeth A. Newell

**Affiliations:** 1) Medical Scientist Training Program, University of Iowa, Iowa City, IA, USA.; 2) Department of Pediatrics, University of Iowa, Iowa City, IA, USA.

**Keywords:** microglia, type I interferon, traumatic brain injury

## Abstract

Type I interferon (IFN-I) signaling has emerged as a central regulator of neuroinflammation across diverse central nervous system disorders, including traumatic brain injury (TBI). While TBI is a leading cause of neurologic morbidity and mortality through young adulthood, there is a paucity of neuroprotective therapies available to clinicians. Recent work has demonstrated neuroprotection after global IFN-I deficiency, yet the cell-type-specific contributions to traumatic brain injury (TBI) and the mechanisms of immune modulation remain poorly defined. Using mice with microglia-specific IFN-I receptor deficiency, we show that loss of microglial IFN-I responsiveness suppresses microglial reactivity, reducing microglial accumulation, synaptic engulfment, antigen presentation, and T cell interactions after TBI. This attenuation preserves neuronal integrity and limits thalamic neuronal loss. Despite this neuroprotection, microglia-restricted IFN-I blockade reveals functional redundancy across CNS cell types, underscoring the multi-cellular nature of IFN-I signaling in the injured brain. Together, our findings delineate a microglial IFN-I–dependent pathway that exacerbates secondary injury after TBI and highlight both the therapeutic potential and inherent limitations of cell-type-targeted IFN-I modulation.

## Introduction

Traumatic brain injury (TBI) is a leading cause of disability and death through young adulthood. People who survive their TBI are often left with lifelong neurologic dysfunction and are more likely to develop dementia and secondary neurodegenerative diseases such as Alzheimer’s or Parkinson’s disease [1–4]. Despite the high morbidity and mortality of TBI, there are currently no neuroprotective therapies to treat TBI. Following TBI, cell damage and death at the site of injury triggers acute immune reactivity including activation of resident glial cells, infiltration of peripheral leukocytes, and increased production of neuroinflammatory mediators. While the initial surge in immune activation may facilitate debris clearance and axonal regeneration, persistent dysregulated immune activation can cause progressive neurodegeneration [5, 6]. Identifying therapeutic targets for TBI therefore requires a deeper understanding of the mechanisms that underpin the cell–mediated neuroimmune response to injury.

An important contributor to neuroinflammation following TBI is the type I interferon (IFN-I) pathway. Although classically known for its role in the antiviral response, the IFN-I pathway is also upregulated in neurodegenerative diseases [7–9]. In both human and experimental TBI, the IFN-I pathway is activated in the brain, and in experimental studies where time course studies are possible, this increase in IFN-I signaling can persist for months after injury [10, 11]. IFN-I pathway-stimulated genes (ISGs) contribute to inflammation, chemokine production, and pro-apoptotic responses. The persistent upregulation of the IFN-I pathway in both brain tissue and isolated immune cells suggests that type I IFNs are a key mechanism of dysregulated immune activation following TBI.

Recent work has demonstrated that IFN-I deficiency or pathway blockade confers neuroprotection following traumatic brain injury [12–14]. However, because multiple cell types express the IFN-I receptor, it remains unclear which cell types drive the observed neuroprotective effect-and whether the benefit of global IFN-I blockade requires coordinated inhibition of IFN-I activity across multiple CNS populations. We have previously shown that there are substantial changes in expression of many IFN-I pathway genes in microglia following TBI, a finding corroborated across several neurodegenerative conditions [7, 9, 15, 16].

In this study we sought to determine the impact of type I interferon signaling in microglia on TBI outcomes. We studied mice with a microglial-cell-specific loss of functional IFN-I receptor (IFNAR) in a widely validated murine model of TBI, the lateral fluid percussion injury model. We performed regional analysis of neuroimmune activation, including a specific focus on the thalamus, given the region’s dense accumulation of both microglia and astrocytes after TBI. We found that microglial-specific IFNAR deficiency reduced thalamic neuropathology after TBI-including decreased microglial accumulation, reduced transcriptional activation, and preservation of neurons-while also altering microglial antigen presentation, T cell engagement, and synaptic protein phagocytosis. Despite these striking changes, elimination of IFNAR-dependent signaling did not prevent TBI-induced spatial memory impairment. These findings underscore the importance of IFN-I signaling in TBI pathophysiology and identify microglia-specific effects on immune cell activation and neuropathology after TBI. Our findings also reveal an inherent redundancy in the TBI-induced IFN-I response, as well as multicellular regulation of this response, critical discoveries when considering new approaches for therapeutic manipulation.

## Materials and methods

### Animals

To generate microglia-specific, inducible Ifnar1 knockout mice, the Jackson laboratory crossed B6.129P2(C)-*Cx3cr1*^*tm2.1 (cre/ERT2)Jung*^/J (Cx3cr1CreER, Stock #020940) with B6(Cg)-*Ifnar1*^*tm1.1Ees*^/J (Ifnar1fl, Stock #028256) mice [17]. The Ifnar1 floxed allele contains loxP sites that flank exon 3 of the Ifnar1 gene. Cre recombinase mediated deletion of exon 3 generates a null allele and non-functional protein. Breeding was done to generate Cx3cr1/Ifnar1fl homozygous and Cx3cr1CreER heterozygous/Ifnar1fl homozygous mice. Mice were housed in mixed-genotype groups. Once mice reached adulthood (greater than 2 months of age), all mice received 100 ul intraperitoneal injections of tamoxifen (dissolved in corn oil; 20mg/mL) every 24 hours for 5 consecutive days. Peripheral *Cx3cr1*- expressing cells were allowed to repopulate for a minimum of 4 weeks, after which Cx3cr1/Ifnar1 fl homozygous mice were referred to as WT^tg^ and Cx3cr1CreER heterozygous/Ifnar1fl homozygous mice were referred to as MG IFNAR KO. Mice were housed in the Animal Care Facility at the University of Iowa (Iowa City, IA) with ad libitum access to food and water. All studies were conducted on adult, 3–6-month-old, male and female mice. The average weight on day of craniectomy was 23.4g ± 2.7g for female mice and 31.1g ± 3.7g for male mice. After craniectomy, mice remained singly caged throughout the duration of the experiments. All procedures performed in this study were done following protocols approved by the Institutional Animal Care and Use Committee at the University of Iowa.

### Fluid Percussion Injury

We induced experimental traumatic brain injury using a lateral fluid percussion injury protocol, as previously described [18]. One day prior to injury, mice received craniectomy to allow for injury against the exposed dura. For this procedure, all mice were anesthetized with a ketamine/xylazine mix (87 mg/kg ketamine and 12 mg/kg xylazine) via intraperitoneal injection. Once under deep anesthesia, mice were mounted in a stereotaxic head frame and the scalp was reflected via a midline incision. A 3-mm OD handheld trephine (University of Pennsylvania Machine Shop) was used for craniectomy on the left parietal skull bone centered between lambda and bregma sutures and between the lateral skull edge and sagittal suture. A 20G needle was used to cut a Luer-lock hub, this hub was secured over the craniectomy site with cyanoacrylate glue (Loctite 760355) and dental cement (Jet Acrylic Liquid mixed with Perm Reline/Repair Resin). The hub was filled with sterile saline and was topped with a sterile intravenous cap to prevent exposure to the environment.

The next day mice received the FPI. The pendulum angle of the FPI device was adjusted before each experiment to ensure that the peak pressure was between 1.3 and 1.5 atmospheres (atm). To achieve this peak pressure, the pendulum angle was varied between 11 and 12 degrees. Prior to injury, mice were anesthetized with 3% inhaled isoflurane, the hub cap was removed, and any air bubbles were flushed out of the hub with sterile saline. Mice were quickly attached to the FPI device by connecting the skull hub to IV tubing that extends out of the fluid percussion device. Mice were laid on their right side and the pendulum was released generating a pressurized fluid pulse against the exposed dura. The duration and peak pressure of the fluid pulse was measured with an oscilloscope (TDS460A). Mice were detached from the device and placed on their backs to measure the amount of time it took for each subject to right themselves. After righting, mice were anesthetized again with 3% inhaled isoflurane, the hub was removed, and the scalp incision was sutured closed. Mice were allowed to recover in a heated cage until ambulatory. To target moderate to severe traumatic brain injury, mice were included if there was gross evidence of cortical disruption and if their righting reflex was >4 minutes. Across all studies, the average righting time ± SD was 391 ± 82 seconds, which corresponded to an average peak pressure of 1.45 ± 0.06 ATM. For all experiments, controls received anesthesia and analgesia without craniectomy or injury.

### RNAscope, Immunohistochemistry

Mice were anesthetized with a lethal dose of ketamine/xylazine and perfused transcardially with ice cold 1X PBS followed by 4% PFA. Brains were extracted and post-fixed in 4% PFA at 4°C for 48 hours before starting a sucrose cryoprotection gradient of 10%/20%/30% sucrose sequentially waiting until the brain had sunk to the bottom of the vessel before proceeding with the next concentration. Brains were embedded in Optimal Cutting Temperature Compound (Tissue-Tek) and 10-micron coronal slices were sectioned. Prior to staining, each slide was baked for 30–60 minutes until dry at 40°C. For RNAscope staining, slides were stained using the manufacturer’s specifications (Table 1). For all immunohistochemistry, sections were washed with 1X PBS and blocked for 1 hour at room temperature (0.5% Triton X-100 and 10% goat serum in 1× PBS). After blocking, sections were incubated overnight in primary antibody diluted in blocking solution. The next day, sections were washed with 1X PBS and proceeded to secondary staining diluted in blocking solution for 1 hour at room temperature. Sections were washed in 1X PBS and mounted with DAPI hardset Vectashield mounting media (H-1500–10). All brain slices were imaged using a slide-scanning microscope (Olympus VS120). Relative occupancy was calculated by measuring the area of the RNAscope *gene of interest* divided by the total area of *Hexb* staining and multiplying this value by 100.

### Nanostring

Mice were euthanized with isoflurane followed by swift regional dissection 7 days following TBI. A circular 2mm Rapid-Punch (EMS #69033–20) was used to isolate ipsilesional thalamic biopsies from the dorsal aspect of the thalamus immediately underlying the hippocampus. Tissue samples were snap frozen with liquid nitrogen and stored at −80 degrees C. RNA was extracted using the Trizol method. The resulting RNA’s quality and quantity were evaluated with the Agilent 2100 Bioanalyzer. Gene expression was normalized and quantified using the NanoString nCounter Neuroinflammation and Host response panels. Data was analyzed using the Rosalind Bio nSolver Advanced Analysis software. Adjustment of p-values to account for multiple testing was performed using the Benamini-Hochberg false discovery rate method with a p<0.05 considered significant. Each Nanostring panel was analyzed separately, and the resulting differentially expressed genes combined into one list for simplicity.

### Flow Cytometry

Mice were euthanized with isoflurane and the left (ipsilesional) forebrain was swiftly dissected and then gently disrupted by a loose fitting dounce homogenization in 5mL of ice-cold HBSS+ (HBSS, 0.5% BSA, 2mM EDTA). After all the brains were homogenized, samples were centrifuged at 310g for 5 minutes; all centrifugation was performed at 4C. The pellet was resuspended in 1mL of HBSS+ via gentle repetitive trituration with a P1000 pipette and transferred to a chilled 2mL Eppendorf tube. Cells were centrifuged for 15s at 100g, and the supernatant containing dissociated cells was transferred to a prechilled 15mL conical tube. The resuspension and short spin was repeated 4 times or until most of the cells were dissociated leaving a small pellet to discard. The suspended cells were passed through a prewetted 40um cell strainer (CLS352340) into an ice-cold 50mL conical tube and pelleted. For myelin removal, cell pellets were resuspended in 30% Percoll^™^ (diluted in HBSS) and centrifuged at 310g for 20 minutes using a slow deceleration. The myelin layer and supernatant were aspirated, and the pellet was washed with 2mL of HBSS and transferred to a FACS tube. Cells were pelleted and then stained. For all staining panels, the cells were first incubated at 4C in 50 ul HBSS+ containing 1ul of Mouse Fc Block for 10 minutes. After blocking, 50 ul of the cell staining cocktail was added (Table 2) and staining was done for an additional 20 minutes at 4C in the dark. After staining, the cells were washed and resuspended in 200–500ul of HBSS+. Single cell suspensions were analyzed either by fluorescence activated cell sorting (FACS) or quantitative flow cytometry. FACS was performed at 4° on a BD Aria II using the 100-μm nozzle at a PSI of 20. The cells were sorted into 2-mL Eppendorf tubes containing 200 μL HBSS+. The tubes containing HBSS+ were vortexed prior to sorting to optimize the pelleting of sorted cells. Quantitative flow analysis was performed using the Cytek Aurora or Cytek Amnis ImageStreamX MkII to obtain flow cytometry imaging. All flow data was analyzed using FlowJo software (version 10.9).

### Single Cell Sequencing and Bioinformatics

Live CD45+ flow-sorted cells were prepared and processed by the University of Iowa Institute of Human Genetics for barcoding using the Chromium Single Cell 3’ reagent kit (10x Genomics). Library preparation was conducted as per the manufacturer’s protocol. Barcoded libraries were sequenced on an Illumina NovaSeq 6000. cDNA libraries from the samples were qualitatively checked and passed assessment. The FASTQ files were generated with bcl2fastq software (Illumina, version). The Cell Ranger analysis pipeline was used to perform alignment to the reference genome, demultiplexing, cell barcode identification, and [insert a verb, e.g. “and creating the gene-cell expression matrices] gene-cell expression matrices. The data were imported into the R/Seurat (R version 4.3.2, Seruate_5.0.1) environment and per-sample Seurat objects were created. After manual review, each sample was filtered for cells with <15% mitochondrial genome and an RNA feature count of >150 and <6600. The 13 different experimental subjects (each from a different mouse) were integrated using the ‘rpca’ rapid integration method (https://satijalab.org/seurat/articles/integration_rpca.html). Integrated data was re-scaled, and PCA and UMAP reductions were calculated. Neighbor-finding and clustering were computed with a cluster resolution of 0.1. Cell-type markers were plotted with ‘VlnPlot’ to determine global cluster identities. Cell type assignments were confirmed based on marker genes from the literature and expert knowledge. Next, the data were grouped by cell identity and converted to loupe files for subsequent analysis in Loupe Browser. For both the microglial and T cell/NK datasets, we used a PCA of 50 to produce unbiased clustering. Differential gene expression (DEG) analysis was also performed in Loupe Browser v.8; DEGs reported had a p-value of <0.05 adjusted for multiple testing using the Benjamini-Hochberg procedure to control FDR.

### Phagocytosis assay

To study microglial phagocytosis, we used the flow cytometry-based toolkit for application of the microglial engulfment of synaptic and myelin proteins (FEAST) protocol, as previously described [19]. Briefly, mice were euthanized with isoflurane and brains were swiftly removed. Ipsilesional or left forebrains were dissected and placed into a glass petri dish, where they were finely minced with a razor blade in ice cold HBSS. Cells were transferred to a 15mL conical and suspended in RPMI-1640 without phenol red + 5mM HEPES (RPMI-H). All centrifugation was performed in a 4 C swinging bucket centrifuge at 310g unless otherwise specified. Cells were pelleted and resuspended in 2 mL of RPMI-H + inhibitor cocktail (1:1000) and incubated for 20 minutes agitating at 4 C. After incubation, cells were pelleted and resuspended in 2 mL of pre-heated digestion cocktail and incubated for 45 minutes at 37 C while gently agitating (Table 3). Cells were pelleted and for the rest of the protocol kept at 4 C. 1mL of chilled FACS buffer was added to stop the digestion and cells were triturated with a P1000 pipette until fully homogenized. The suspension was filtered through a 70 um filter (CLS352350) into a 50 mL tube pre-coated with FACS buffer. To ensure maximal yield, each tube was rinsed with 2mL FACS buffer and filtered through the same 70 um filter. Samples were pelleted, resuspended in 6 mL of 30% Percoll^™^ and spun at 310g for 2 minutes with the deceleration set to 2 to avoid disruption of the myelin layer. The myelin layer was aspirated and cells were resuspended in 3 mL of HBSS, transferred to FACS tubes and pelleted. Cells were resuspended in 50 μl of HBSS+ 2mM EDTA with FcR block and fixable blue LIVE/DEAD viability dye for 20 minutes on ice. 50ul of staining cocktail containing CD11b and CD45 diluted in FACS buffer (Table 2) was added and incubated for an additional 20 minutes at 4 C. Cells were washed with 200 ul of FACS buffer, pelleted and fixed with 200ul of fixation buffer for 20 minutes at room temperature. After fixation, each sample was split into 2 tubes, one for intracellular staining of MBP and SNAP25, and the other for staining with isotype controls. The rest of the protocol was performed at room temperature. Cells were permeabilized with 200 ul of permeabilization buffer and spun at 500g for 5 minutes. Buffer was decanted and an additional 50 ul of permeabilization buffer with 10% goat serum was added to each sample and incubated for 20 minutes. Then 50 ul of permeabilization buffer with antibodies for intracellular staining was added and incubated for an additional 20 minutes. (Antibody dilutions are listed in Table 2). Cells were washed in 200 ul permeabilization buffer, pelleted and washed with 200ul FACS buffer. The resulting pellet was resuspended in 250 ul of FACS buffer and proceeded to flow cytometry on the Aurora machine. Flow data was analyzed with Flowjo.

### Barnes Maze

Learning and memory were assessed using the Barnes Maze. The Barnes Maze (SD instruments 7001–0235) was a circular table with a 36 in diameter surrounded by 20 equally spaced circular holes. Prior to the training trials on day 1 of testing, each mouse was placed in the maze with aversive stimuli off and spatial cues covered. After 1 minute of habituation, each subject was placed in the escape box for 2 minutes before returning to their home cage. After habituation, the training phase commenced. Aversive stimuli (two 150 W lights + small fan) were turned on at the start of the acquisition trials. Each mouse performed 4 trials per day for 4 days, and every trial was recorded and analyzed with a mounted camera and Anymaze software (version x). For each trial, a single mouse was placed in the center of the maze, enclosed within the starting box to ensure random facing, when the timer started the starting box was lifted and the mouse was allowed to search for the escape box. The trial ended after 80 seconds, or when the mouse entered the escape box. At the end of the trial each mouse remained in the escape box for 30 seconds before being returned to their home cage. The probe trial occurred on day 5 of testing. The probe trial consisted of a 60 second trial where the escape box was removed, and memory of the escape hole location was tested. At the end of 60 seconds, the mice were returned to their home cage.

### Statistical analysis

Statistical analysis was performed using Prism 11 (GraphPad Software) except for single cell RNA sequencing and Nanostring analysis which are described in detail above. Distribution normality was determined using the Shapiro-Wilk test. For experiments containing two groups, analysis was done using a two-tailed, unpaired t-test for normally distributed data or the Mann-Whitney *U* test for non-normally distributed data. For experiments utilizing greater than two experimental groups, statistical analysis was done using one-way ANOVA. If one-way ANOVA revealed significant differences, post-hoc multiple comparisons were done by Sidak’s test. For multiday Barnes maze testing, a two-way repeated measures ANOVA was done evaluating the impact of genotype and injury group across time. This was followed by Tukey’s test for post-hoc multiple comparisons if significant effects existed in the interaction or main effects. A value of p<0.05 was considered statistically significant. Comprehensive results of statistical analysis for all figures are provided in Supplemental Table 4. All data are displayed as mean ± SEM. WT transgenic and microglial IFNAR KO mice were randomly assigned to control or TBI groups. All testers were blinded to genotype and injury status. Results were combined from multiple TBI experiments for each of the studied endpoints to demonstrate reproducibility.

## Results

**Figure 1:**
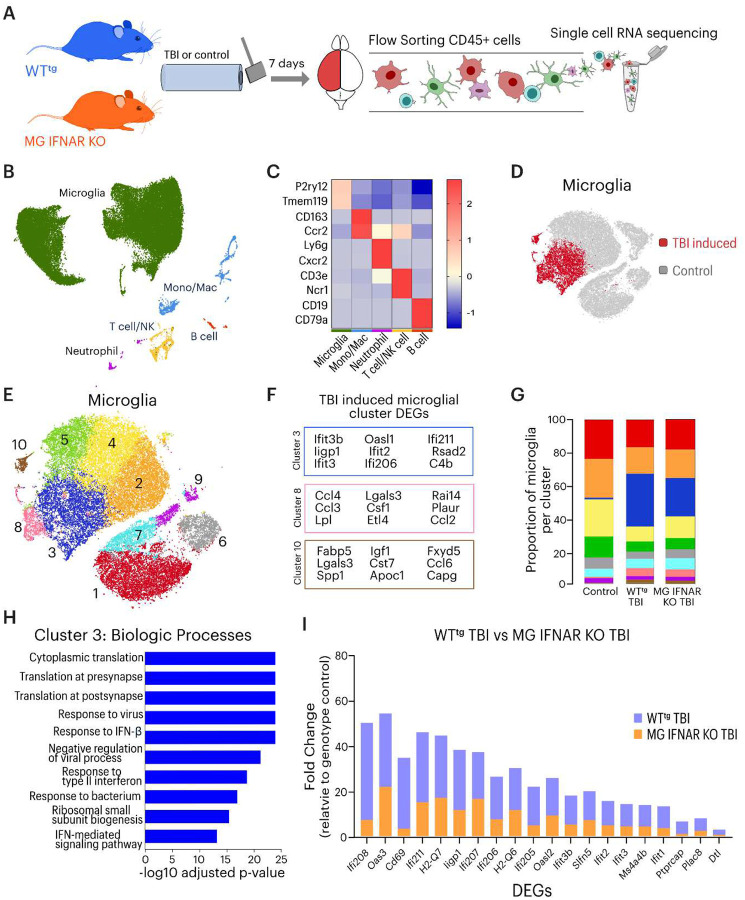
Microglial IFNAR deficiency reduced interferon-responsive microglia without alteration of other TBI-induced subsets. **(A)** Schematic of experimental paradigm for single cell RNA sequencing. **(B)** t-distributed Stochastic Neighbor Embedding (t-SNE) plot of all CD45+ cells sequenced; total of 42,771 cells, colored by cell type. **(C)** Heatmap of cell type marker genes. **(D)** t-SNE plot showing all microglial cells sequenced with the TBI-induced microglial clusters highlighted in red. **(E)** t-SNE plot of microglia cells colored by cluster. **(F)** Select differentially expressed genes (DEGs) in TBI-induced microglial clusters. **(G)** Proportion of microglial cells in each cluster per condition. **(H)** Gene ontology analysis of cluster 3 DEGs. **(I)** Pseudobulk analysis of all WT^tg^ TBI microglia versus all MG IFNAR KO TBI microglia. Stacked bar graphs show significantly differentially expressed genes and are plotted with values relative to genotype control. n=3 WT^tg^ control, n=3 MG IFNAR KO control, n=4 WT^tg^ TBI, n=3 MG IFNAR KO TBI. Significance determined by a p <0.05 adjusted for multiple testing using the Benjamini-Hochberg procedure.

To determine if microglial-specific IFN-I/IFNAR signaling is a major driver of pathophysiology after TBI, we used male and female Cx3cr1 CreER +/−; IFNAR1 flox/flox or Cx3cr1 CreER −/−; IFNAR1 flox/flox mice to generate a microglia-specific IFNAR KO (MG IFNAR KO) or transgenic mice with wild-type IFNAR function (WT^tg^, genotype Cx3cr1 CreER −/−; IFNAR1 flox/flox) (Supplemental Fig 1) [20]. We first sought to evaluate the impact of IFN-I signaling in microglia on brain immune cell populations and their transcriptomes following TBI. We employed single-cell sequencing at seven days after experimental TBI, comparing to non-surgical controls. To enrich for microglia and other leukocytes, CD45+ cells were collected using FACS prior to single cell RNA-sequencing (Fig 1A). A total of 42,771 cells met inclusion criteria; the expected CD45+ cell types (microglia, monocyte/macrophage, T cells, natural killer cells, neutrophils, and B cells) were observed using unbiased clustering (Fig 1B) followed by identification with known cell markers (Fig 1C). As expected, microglia were the most abundant cell type representing 91.8% of the total cells sequenced with 39,265 microglial cells sequenced. TBI-induced changes in the proportion of cell-types detected were modest. WT^tg^ TBI mice showed a trend towards a reduced percentage of microglia, although still accounting for 87% of all CD45+ cells. In both WT and MG IFNAR KO mice, the percentage of T cell/NK cells increased following TBI (Supplemental Fig 2).

Next, given the known heterogeneity of microglia, we performed subclustering of all microglial cells. Unbiased clustering resulted in 10 unique microglial subclusters, 3 of which were specific to TBI (Fig 1D–E). Cluster 3 was the largest TBI-induced cluster with 6,173 cells; differential expression analysis between subclusters revealed that Cluster 3 was highly enriched for known interferon-stimulated genes (*Ifit3, Rsad2, Irf7*) but also included more general immune responsive genes (*C4b, Apoe, Ccl5*). We labeled this cluster as interferon-responsive microglia, which have been previously described after TBI and other neurodegenerative diseases [9, 21, 22]. Cluster 8 had 1,131 cells and was enriched in DEGs relating to chemotaxis (*Ccl4, Ccl3, Ccl2*) and neurodegeneration (*Spp1, Lgals3, Atf3*). Finally, Cluster 10 was the smallest TBI-induced cluster with 946 cells; the DEGs displayed notable overlap with cluster 8, only 127 DEGs were unique to cluster 10 (Fig 1F). The full list of DEGs from all subclusters is available in Supplemental Table 1.

All three TBI-induced clusters were represented in the MG IFNAR KO TBI mice with cluster 8 and 10 showing similar proportionality as in WT^tg^ TBI. Cluster 3, however, was reduced in MG IFNAR KO TBI mice from 32% in WT^tg^ TBI to 23% in MG IFNAR KO TBI (Fig 1G). This reduction, but not elimination, of cluster 3 interferon responsive microglia is consistent with redundancy in the signaling pathways that contribute to induction of interferon stimulated genes (ISGs) [23, 24].

Gene ontology analysis of cluster 3 showed significant enrichment for response to IFNβ, response to type II interferon, and IFN-mediated signaling pathway, thus confirming their identity as interferon responsive microglia (Fig 1H). Lastly, to further investigate how MG IFNAR KO modulates the overall microglial transcriptome, we used pseudobulk DE analysis to compare WT^tg^ TBI microglia to all MG IFNAR KO TBI microglia and found 20 significantly downregulated DEGs (Fig 1I). Each DEG has been recognized as interferon-induced, further demonstrating the selective effect of microglial IFNAR deficiency on the microglial transcriptome following TBI.

Gene ontology analysis of cluster 3 showed significant enrichment for response to IFNβ, response to type II interferon, and IFN-mediated signaling pathway, thus confirming their identity as interferon responsive microglia (Fig 1H). Lastly, to further investigate how MG IFNAR KO modulates the overall microglial transcriptome, we used pseudobulk DE analysis to compare WT^tg^ TBI microglia to all MG IFNAR KO TBI microglia and found 20 significantly downregulated DEGs (Fig 1I). Each DEG has been recognized as interferon-induced, further demonstrating the selective effect of microglial IFNAR deficiency on the microglial transcriptome following TBI.

**Figure 2.**
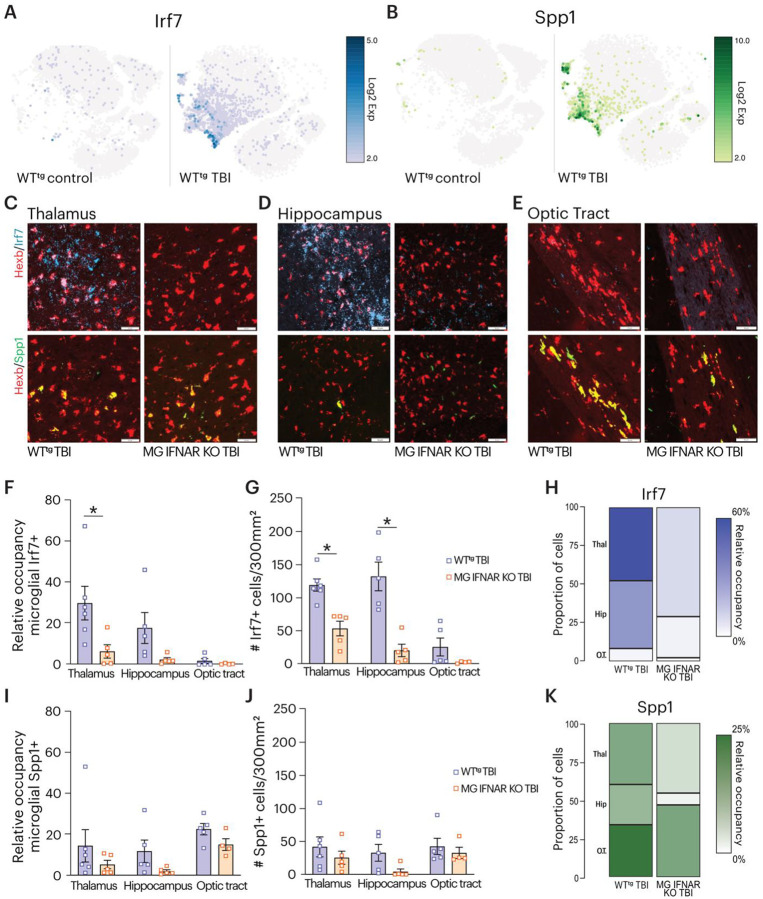
TBI-induced microglial subpopulations show distinct regional distributions and are differentially impacted by MG IFNAR KO. **(A-B)** t-SNE plot of microglial *Irf7* and *Spp1* expression 7 days post WT^tg^ control and WT^tg^ TBI. **(C-E)** Representative images of RNAscope staining 7 days after WT^tg^ or MG IFNAR KO TBI for *Irf7* (top) and *Spp1*(bottom) colocalized with the microglial marker *Hexb* in the thalamus, hippocampus and optic tract. Scale bar = 50um **(F-G)** Regional quantification of microglial *Irf7* relative occupancy and cell counts of *Irf7+* microglia in WT^tg^ and MG IFNAR KO TBI subjects. **(H)** Regional representation of combined proportion and relative occupancy of *Irf7+* microglia. **(I-J)** Regional quantification of microglial *Spp1* relative occupancy and cell counts of *Spp1+* microglia in WT^tg^ and MG IFNAR KO TBI subjects. **(K)** Regional representation of combined proportion and relative occupancy of *Spp1+* microglia. n= 6 WT^tg^ TBI, n=5 MG IFNAR KO. Unpaired t-test * p<0.05.

While ScRNAseq revealed distinct subclusters of microglia, the regional distribution of these microglial populations following TBI is unknown. Subpopulations of reactive microglia have been shown to reside in spatially distinct patterns, which can provide additional clues as to their in vivo function [25]. We used multiplex RNAscope to evaluate the spatial distribution and to confirm the impact of microglial IFNAR signaling on microglial subpopulations in situ. To identify interferon-responsive microglia, we chose the cluster 3 DEG, *Irf7*, a key transcription factor in the interferon pathway (Fig 2A) [26]. For our non-interferon-related, TBI-induced microglial gene marker, we chose *Spp1*. *Spp1* was enriched in clusters 8 and 10 (trauma-induced clusters) and plays a known role in microglial reactivity and phagocytosis after both TBI and other neurologic insults (Fig 2B) [27, 28]. *Hexb* was used as a microglia marker [29]. We quantified *Irf7* or *Spp1* colocalization with *Hexb* in three different regions: thalamus, hippocampus, and optic tract. We have previously identified the thalamus as a key region of subacute and chronic microglial activation [12]. The hippocampus is also an ideal region for this study, given its proximity to the lesion epicenter but without the degree of variable cell loss and necrosis that is seen in the perilesional cortex. Lastly, the optic tract was chosen in order to study a white matter tract with notable microglial activation, but lacking gross tissue disruption from primary injury, as often occurs in the corpus callosum. We quantified both the number of microglia expressing the gene of interest and the relative occupancy of the gene within microglial cells, which is indicative of the amount of RNA transcripts present.

Consistent with our ScRNAseq data, in uninjured control subjects, *Irf7* and *Spp1* microglial expression was minimal; however, after injury, microglial expression of both genes increased in WT^tg^ and MG IFNAR KO mice (Fig 2C–E). WT^tg^ mice showed a TBI-induced increase in both the number of *Irf7*+ microglia and the relative occupancy of *Irf7* in microglia in the thalamus and hippocampus compared to non-injured controls. Nominal *Irf7* expression was observed in the optic tract compared to the other quantified regions. MG IFNAR KO mice showed an injury-induced increase in *Irf7*+ microglia in the thalamus and hippocampus. However, compared to WT^tg^ TBI mice, MG IFNAR KO TBI mice had decreased number of *Irf7*+ microglia and relative occupancy of microglial *Irf7* in both the thalamus and hippocampus (Fig 2F–G).

*Spp1*+ microglia were increased in the thalamus, hippocampus, and optic tract after TBI in both WT^tg^ and MG IFNAR KO mice (Fig 2I–J). Compared to *Irf7*+ microglia, there were fewer *Spp1*+ microglia and unlike *Irf7*, the highest relative occupancy of *Spp1* microglial expression was localized to the optic tract (Fig 2I). MG IFNAR KO did not significantly reduce the number of *Spp1*+ microglia or *Spp1* relative occupancy in any of the regions studied (Fig 2I–J). Altogether, in-situ hybridization showed regional differences in where microglia subpopulations accumulate after TBI. Additionally, in concordance with our single cell sequencing data, MG IFNAR KO selectively reduced *Irf7* expression and accumulation without modulating *Spp1+* microglial populations following TBI.

We next wanted to determine if the alterations in the microglial transcriptome and subpopulations were sufficient to have broader impacts on the neuroimmune response to TBI. We first addressed this question by using NanoString multiplex assays to determine the impact of microglial IFNAR deficiency on tissue level gene expression. We performed punch biopsies of the dorsal thalamus, a region with a robust secondary neuroimmune response, seven days after TBI. We utilized the Neuroinflammation and Host Response NanoString panels to interrogate the expression of 1,320 genes.

**Figure 3.**
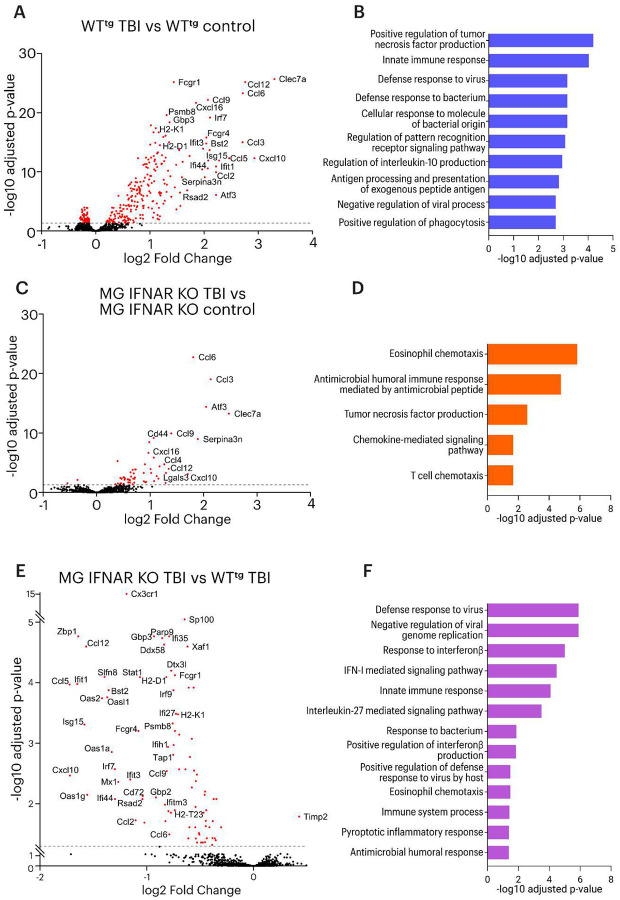
Microglial IFNAR deficiency reduces neuroinflammatory thalamic gene expression following TBI. **(A)** Volcano plot of WT^tg^ TBI vs. WT^tg^ control mice. Red dots are significantly differentially expressed genes. **(C)** Volcano plot of MG IFNAR KO TBI vs MG IFNAR KO control. **(E)** Volcano plot of MG IFNAR KO TBI vs WT^tg^ TBI mice. All dots above the grey dotted line are significantly differentially expressed genes. n=5 WT^tg^ control, n= 7 WT^tg^ TBI, n=4 MG IFNAR KO control, n=4 MG IFNAR KO TBI. Significantly differentially expressed genes determined by a p <0.05 adjusted for multiple testing using the Benjamini-Hochberg procedure. Most significantly differentially expressed biologic processes for **(B)** WT^tg^ TBI vs. WT^tg^ control, **(D)** MG IFNAR KO TBI vs MG IFNAR KO control, and **(F)** MG IFNAR KO TBI vs WT^tg^ TBI. Significant biologic processes were determined with iPathway guide’s smallest common denominator pruning with a p-adj of <0.05.

After WT^tg^ TBI, there were 166 upregulated, differentially expressed genes in the thalamic tissue (FC>0.6, p<0.05) compared to WT^tg^ control (Fig. 3A). Gene ontology analysis was performed and identified numerous significantly upregulated biologic processes related to immune responses (Fig. 3B). When comparing MG IFNAR KO TBI mice to MG IFNAR KO uninjured controls, there were significantly fewer differentially expressed genes and enriched biologic pathways (Fig. 3C, D). Overall, there were only 40 upregulated DEGs with GO analysis identifying enrichment for genes related to chemotaxis. Finally, we compared MG IFNAR KO TBI to WT^tg^ TBI (Fig. 3E, F); we found 54 downregulated DEGs in the MG IFNAR KO compared to WT^tg^ TBI mice. Gene ontology revealed that the downregulated genes in the MG IFNAR KO TBI mice represented key biologic processes related to viral defense response, type I interferon production and response, and pyroptosis-mediated inflammation. Complete lists of DEGs and GO analysis results are available in Supplemental Tables 2 and 3 respectively. In summary, the NanoString results reveal that the robust transcriptional immune response to TBI within the thalamus is dramatically attenuated when IFNAR is knocked out in microglia.

**Figure 4.**
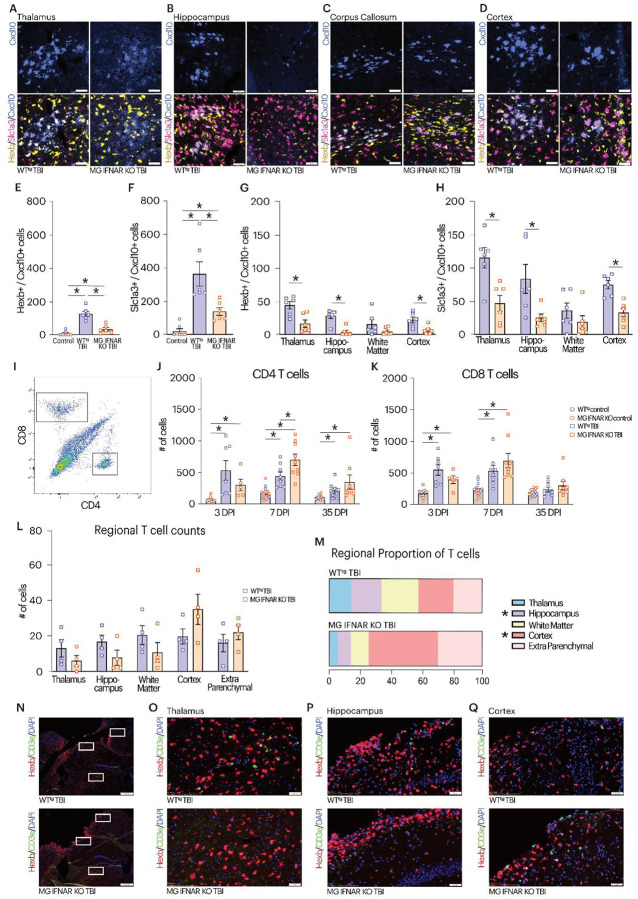
Microglial IFNAR deficiency attenuates glial *Cxcl10* expression and alters the regional accumulation of T cells following TBI. **(A-D)** Representative RNAscope images of *Cxcl10* colocalized with *Hexb* or *Slc1a3* 7 days after WT^tg^ or MG IFNAR KO TBI. Scale bar = 50 um **(E)** Quantification of total *Cxcl10*+ microglia. **(F)** Quantification of total *Cxcl10*+ astrocytes. **(G)** Quantification of *Cxcl10*+ microglia by region. **(H)** Quantification of *Cxcl10*+ astrocytes by region. n=7 controls, n=6 WT^tg^ TBI, n= 6 MG IFNAR KO TBI. **(I)** Representative flow cytometry gating of CD4 and CD8 T cells. **(J-K)** Quantification of CD4 T cells and CD8 T cells at 3-, 7- and 35-days post injury in the ipsilesional forebrain. n = 11–18 controls, n=7–9 WT^tg^ TBI, n= 5–9 MG KO TBI. **(L)** Quantification of CD3e T cells 7 days after TBI. **(M)** Regional proportion of T cells in WT^tg^ TBI or MG IFNAR KO TBI 7 days after injury. n= 4 WT^tg^ TBI, n=4 MG IFNAR KO TBI. **(N-Q)** Representative RNAscope images of *Hexb, CD3e*, and DAPI, scale bar = 500 um **(N)** and 50 um **(O-Q)**. One-way ANOVA with Sidak’s multiple comparisons test **(E, F, J, and K)** or un-paired T test **(G, H, and L)** *p<0.05.

After confirming that microglial-specific IFNAR KO was sufficient to alter the tissue-level gene expression response to TBI, we next wanted to evaluate the impact of microglial IFN-I signaling on the T cell response to TBI. A key function of type I interferon signaling is recruitment and activation of effector cells, including T cells. We previously showed that in mice globally deficient in IFNAR signaling, TBI-induced expression of the chemokine *Cxcl10* was reduced and this was associated with decreased accumulation of T cells in the brain at 3- and 10-days post-injury [12]. In TBI and in ageing-induced neurodegeneration, others have also shown a critical role for CXCL10 in the accumulation of CD8+ T cells in the brain, with both microglia and astrocytes identified as the cellular source of CXCL10 [14, 30]. In our present work, NanoString data indicated that TBI-induced *Cxcl10* expression was reduced in MG IFNAR KO vs WT^tg^ mice following FPI (Fig 3E). We next sought to evaluate if microglial-specific IFNAR deficiency affected *Cxcl10* expression in both microglia and astrocytes. We performed multiplex RNAscope staining for *Cxcl10*, *Hexb* (microglial marker), and *Slc1a3* (astrocyte marker) seven days post injury in WT^tg^ and MG IFNAR KO mice (Fig 4A–C) [31]. *Cxcl10* staining was largely absent from uninjured control brain sections (unpictured). After TBI, several regions of the brain were enriched for *Cxcl10* expression including the thalamus, hippocampus, white matter tracts, and cortex. Although both genotypes showed a TBI-induced increase in the total number of *Cxcl10+* microglia and astrocytes, microglial-specific IFNAR deficiency resulted in a relative reduction in *Cxcl10+* microglia and astrocytes across multiple regions (Fig 4E–H). Of note, astrocytes were a more substantial cellular source of TBI-induced *Cxcl10* expression, with greater numbers of *Cxcl10*+ astrocytes than *Cxcl10+* microglia, following TBI. While microglial-specific IFNAR KO did decrease astrocyte *Cxcl10* expression, the reduction was greater in microglia (Fig 4 E–F).

Following our evaluation of *Cxcl10* expression, we next turned to assess the impact of microglial-IFNAR deficiency on T cell accumulation in the brain via flow cytometry and multiplex RNAscope. Using flow cytometry, we quantified both CD4 and CD8 T cells within the ipsilesional forebrain at 3-, 7-, and 35-days post injury in WT^tg^ and MG IFNAR KO mice (Fig 4H). CD4 T cells were increased after TBI in both genotypes at all timepoints measured. In fact, MG IFNAR KO TBI mice showed an increase in the number of CD4 T cells at seven days post-injury compared to WT^tg^ TBI mice (Fig 4I). CD8 T cells were also increased in both WT^tg^ and MG IFNAR KO mice at three- and seven-days post-injury but were not different than controls at thirty-five days post-injury (Fig 4J). Next, using RNAscope, we stained for *CD3e*, a pan T cell marker, and *CD8a* to study where T cells were accumulating within the brain at seven days post-injury (Fig 4K). In agreement with our flow cytometry data, we observed an injury-induced increase in T cells for both WT^tg^ and MG IFNAR KO TBI mice. We were surprised, however, to observe regional differences in where the T cells accumulated. For WT^tg^ TBI mice, T cells were distributed across the thalamus, hippocampus, white matter, cortex, and extra parenchymal space (dura, ventricles) (Fig 4L). In MG IFNAR KO TBI mice, there was decreased accumulation of T cells within subcortical regions such as the thalamus and hippocampus while there was an increased number of T cells accumulating in the cortex and extra parenchymal space, when compared to WT^tg^ TBI (Fig 4M). In summary, the two assays evaluating T cell accumulation showed TBI-induced increases in both genotypes, but regional differences in the location of accumulation.

**Figure 5.**
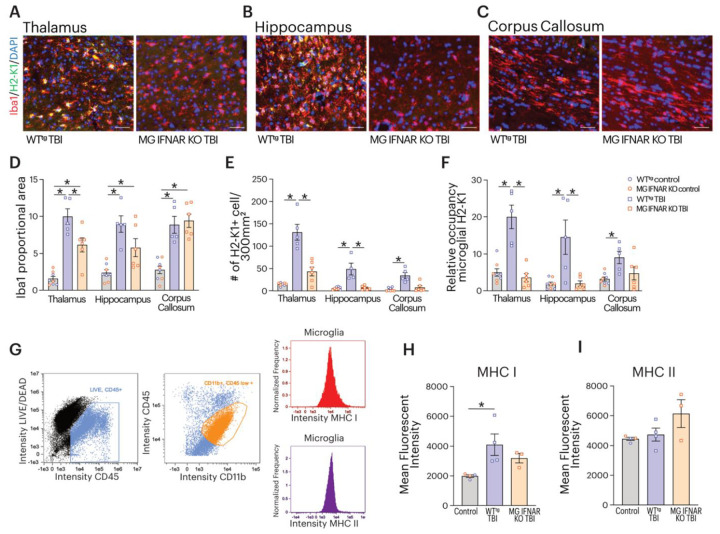
Microglial IFNAR deficiency reduces TBI-induced MHC class I expression. **(A-C)** TBI-induced microglial *H2-K1* expression using dual RNAscope and IHC 7 days post WT^tg^ or MG IFNAR KO TBI in the thalamus, hippocampus, and corpus callosum. Scale bar = 50 um **(D)** Proportional area of IBA1+ staining by region. **(E)** Regional cell counts of *H2-K1+* microglia. **(F)** Regional quantification of microglial *H2-K1* relative occupancy. n=8 controls, n= 5 WT^tg^ TBI, n=6 MG IFNAR KO. **(G)** Representative flow gating for microglial MHC I and MHC II staining. **(H-I)** Quantification of the mean fluorescent intensity of MHC I or MHC II. n=4 controls, n= 4 WT^tg^ TBI, n=3 MG IFNAR KO. One-way ANOVA with Sidak’s multiple comparisons test (**D, E, F, H, and I)** *p<0.05.

In addition to potential roles in recruitment and expansion of T cells in the brain, microglia also play a critical role in the activation of T cells through MHC-dependent antigen presentation [32–34]. Our prior work demonstrated that global deficiency of IFNAR prevented TBI-induced upregulation of MHC class I genes [12]. We now sought to evaluate if microglial-specific IFNAR deficiency would alter microglial MHC I gene and protein expression and if this was associated with altered microglia -T cell engagement. At the tissue level, our NanoString data revealed robust reduction in genes related to antigen presentation and processing within the thalamus in the setting of microglial INFAR KO (H2-K1, H2-D1, Fig 3E). Because all nucleated cells express MHC I, we next used dual RNAscope immunohistochemistry (IHC) to evaluate for microglial-specific MHC I gene expression. We stained for *H2-K1*, an MHC class I molecule, with RNAscope followed by IHC for the microglial/macrophage marker IBA1 seven days after TBI (Fig 5A–C). We found TBI-induced changes in microglial morphology and accumulation throughout the ipsilateral hemisphere in both genotypes. Compared to WT^tg^ TBI mice, MG IFNAR KO TBI mice had a significant decrease in IBA1-positive cells within thalamus (Fig 5D). Upon assessment of H2-K1 expression, only WT^tg^ TBI mice showed an increase in the number of *H2-K1*+ microglia overall as well as increases in the number of *H2-K1+* microglia within the thalamus, hippocampus, and corpus callosum compared to controls. MG IFNAR KO TBI mice had significantly fewer *H2-K1*+ microglia and decreased relative occupancy of microglial *H2-K1* compared to WT^tg^ TBI mice in the thalamus and hippocampus and were no different from controls (Fig 5E–F). Lastly, we used flow cytometry to label protein expression of MHC I and MHC II on the surface of microglial cells seven days after injury (Fig 5G). As expected, WT^tg^ TBI mice had significantly higher mean fluorescent intensity of MHC I compared to control mice. MG IFNAR KO TBI mice, however, were not significantly different than controls (Fig 5H). Interestingly, neither WT^tg^ nor MG IFNAR KO mice showed a TBI-induced increase in MHC II expression, suggesting that TBI may preferentially increase MHC class I antigen presentation (Fig 5I). Taken together, these data suggest that microglial-specific IFNAR signaling is a driver of microglial-MHC class I expression after TBI.

**Figure 6.**
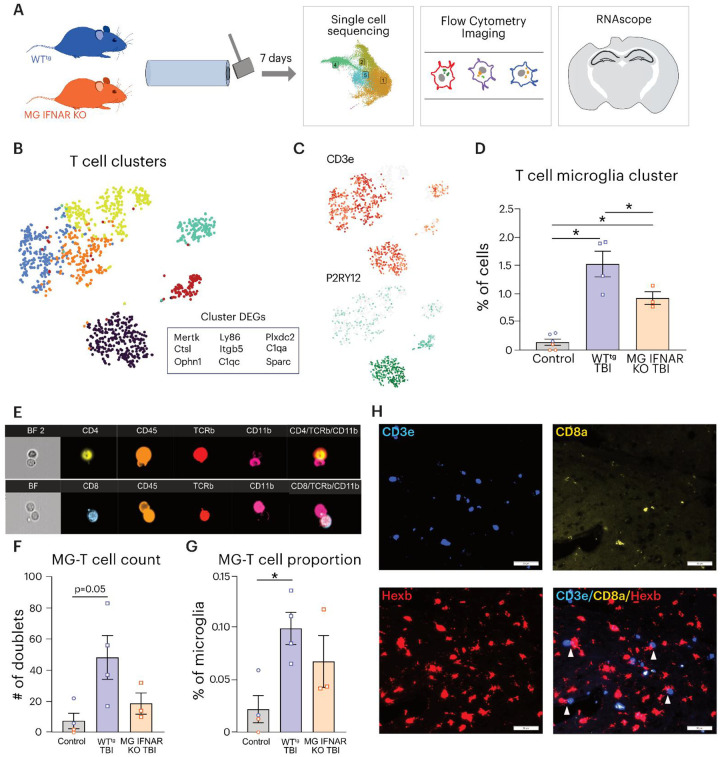
Microglial IFNAR deficiency decreases microglial-T cell interactions after TBI. **(A)** Experimental paradigm showing multiple experimental assays used to identify and quantify microglial-T cell engagement. **(B)** t-SNE plot of T cell clusters and differentially expressed genes for microglia T cell doublet cluster. **(C)** t-SNE plots showing expression of both *CD3e* and *P2ry12* in the doublet cluster. **(D)** Quantification of proportional abundance of doublet cluster. n=6 controls, n=4 WT^tg^ TBI, n=3 MG IFNAR KO TBI. **(E)** Representative flow cytometry images of microglia T cell doublet events. **(F)** Quantification of the flow cytometry doublets. **(G)** Quantification of the proportion of microglia that were T cell doublets. n=4 controls, n=4 WT^tg^ TBI, n=3 MG IFNAR KO TBI. **(H)** RNAscope images of *CD3e* and *CD8a*, and *Hexb* staining 7 days after TBI, white arrowheads showing microglial T cell interaction, scale bar = 50um. One-way ANOVA with Sidak’s test for multiple comparisons *p<0.05.

Next, we sought to evaluate if the decrease in microglial MHC I expression in MG IFNAR KO TBI mice resulted in reduced microglia and T cell engagement (Fig 6A). When examining the DEGs from the largest cluster of T cells (309 cells) in our ScRNAseq experiment, we were surprised to discover that they were highly enriched for genes relating to phagocytosis (*Mertk, C1qc, C1qa*) and were positive for both T cell markers (*CD3e, CD4, CD8a*) and microglia cell markers (*P2ry12, Tmem119*) (Fig 6B–C). Additionally, this cluster was TBI-induced with very few cells in the cluster observed in control mice. We quantified the proportion of cells in the T cell microglia cluster and found that, while both TBI groups had significantly higher proportion of cells in this cluster compared to controls, these cells were significantly less abundant in the MG IFNAR KO TBI compared to the WT^tg^ TBI subjects (Fig 6D). Based on a recent study that determined a similar single cell sequencing cluster in diseased human brain tissue was due to doublet events formed from microglial and T cell interactions, we hypothesized our cluster represents microglial and T cell doublets following TBI [35]. To assess this, we used imaging flow cytometry to evaluate for microglial-T cell conjugates. Indeed, we captured images of microglia forming doublets with both CD4 and CD8 T cells and could quantify these events after TBI (Fig 6E). Both total microglial-T cell doublet counts, and the proportion of microglia engaged with T cells were significantly higher in WT^tg^ TBI mice when compared to uninjured controls. Conversely, microglial-T cell doublets were not increased in MG IFNAR KO mice following TBI (Fig 6F–G). Lastly, we used RNAscope to visualize these microglia-T cell interactions in situ. Seven days post-injury, we found many microglial-T cell interaction events throughout the brain sections, with both *CD3+CD8+* and *CD3+CD8-* T cells engaging with microglia (Fig 6H). In conclusion, we confirmed microglial and T cell interactions after TBI with three different assays and have evidence that these interactions are decreased by microglial specific IFNAR deficiency.

**Figure 7.**
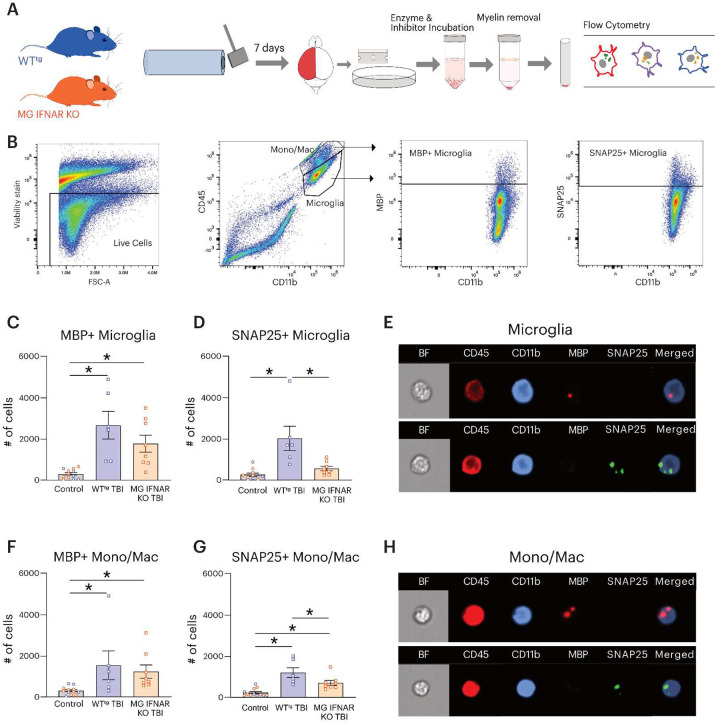
Microglial IFNAR deficiency alters phagocytosis of presynaptic protein after TBI. **(A)** Experimental paradigm of flow-based phagocytosis assay 7 days post-injury in WT^tg^ and MG IFNAR KO mice. **(B)** Flow gating for microglia or monocyte/macrophages (mono/mac) with intracellular MBP+ or SNAP25+ staining. **(C-D)** Quantification of MBP+ or SNAP25+ microglia. **(E)** Flow imaging showing microglia with intracellular MBP or SNAP25 staining. **(F-G)** Quantification of MBP+ or SNAP25+ mono/macs. **(H)** Flow imaging showing monocyte/macrophages with intracellular MBP or SNAP 25 staining. n= 10 controls, n=6 WT^tg^ TBI, n=8 MG IFNAR KO TBI. One-way ANOVA with Sidak’s test for multiple comparisons *p<0.05.

In addition to evaluating how microglial IFNAR signaling impacts coordination of the immune response to TBI, we also sought to determine if neuropathology is altered, including that mediated directly by microglia. A primary function of microglia is phagocytosis, which in the setting of neuroinflammation, may be dysregulated resulting in excessive ingestion of CNS material [36]. We used a published protocol, Flow cytometric Engulfment Assay for Specific Target proteins (FEAST), to interrogate for microglial ingestion of myelin basic protein (MBP) and synaptosomal protein-25 (SNAP25) (Fig 7A–B) [19]. Both WT^tg^ and MG IFNAR KO mice showed an injury-induced increase in the number of microglia positive for intracellular MBP (Fig 7C). Interestingly, only WT^tg^ TBI had increased SNAP25+ microglia, while MG IFNAR KO TBI mice were no different than non-injured controls and had significantly decreased SNAP25+ microglia compared to WT^tg^ TBI counterparts (Fig 7D). We also looked at intracellular staining of MBP and SNAP25 in monocytes and macrophages (mono/mac), since these cells are also responsible for phagocytosis in the brain. We found that mono/macs also showed a TBI-induced increase in the number of cells with intracellular MBP in both WT^tg^ and MG IFNAR KO mice (Fig 7F). Although MG IFNAR KO mono/macs showed a TBI-induced increase in SNAP25 phagocytosis, this injury-induced response was significantly less than the WT^tg^ TBI mice (Fig 7G). These data suggest that while MG IFNAR KO mice mount a similar phagocytic response to MBP, they showed reduced phagocytosis of SNAP25 in both microglia and monocyte/macrophages after TBI.

**Figure 8.**
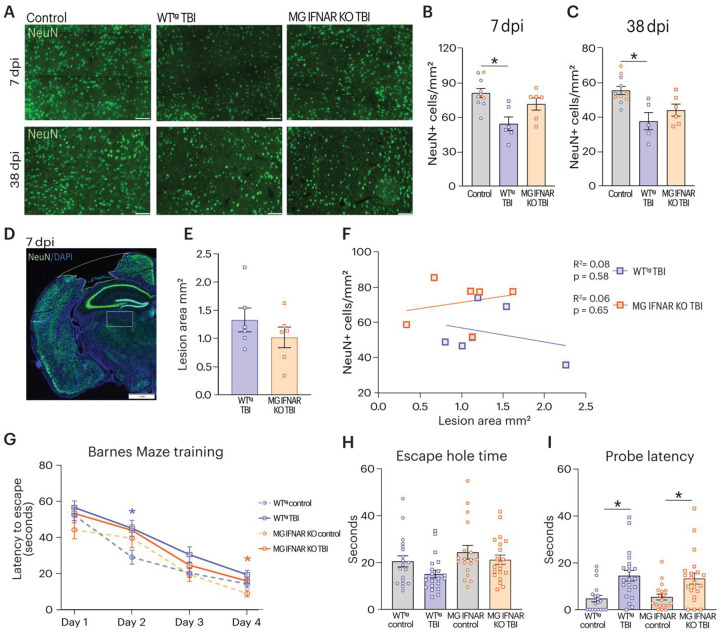
Microglial IFNAR deficiency reduces thalamic neuronal loss, but TBI-induced memory impairment persists. **(A)** Representative images of thalamic NeuN immunohistochemistry at 7 or 38 dpi in control, WT^tg^ TBI, and MG IFNAR KO TBI mice, scale bar = 100um. **(B-C)** Quantification of NeuN+ cells at 7 and 38 dpi. **(D)** Representative image showing cortical lesion area and NeuN thalamic region of interest, scale bar = 1mm. **(E)** Quantification of lesion area in WT^tg^ TBI and MG IFNAR KO TBI 7 dpi. **(F)** Plot demonstrating lack of correlation between lesion area and neuronal loss 7 dpi in WT^tg^ TBI and MG IFNAR KO TBI. Barnes Maze testing was performed 4 weeks post-injury. **(G)** Latency to escape into the escape box was measured daily throughout Barnes Maze training. On day 5 of testing, the escape box was removed and memory of the escape box location was testing during a probe trial. **(H)** Quantification of the amount of time mice spent around the escape hole for the duration of the probe trial. **(I)** Quantification of the latency to the first escape hole entry during the probe trial. One-way ANOVA with Sidak’s multiple comparisons test **(B,C, H, and I)**, Unpaired t-test **(E)**, and Two-way ANOVA with Tukey’s test for multiple comparisons **(G)** *p<0.05.

Next, we sought to assess the impact of microglial IFNAR signaling on neuronal survival. Previously our lab demonstrated decreased neuronal loss following TBI in the setting of global IFNAR deficiency [12]. We wanted to investigate whether microglia-specific IFNAR deficiency was sufficient to recapitulate this finding, particularly given the robust attenuation of the thalamic neuroimmune response in MG IFNAR KO mice. We used NeuN IHC to evaluate neuronal numbers within the ipsilesional dorsal thalamus (Fig 8A). At seven days post-injury, WT^tg^ TBI mice showed the expected decrease in thalamic neurons, while MG IFNAR KO TBI mice were not significantly different from controls (Fig 8B). Similarly, at a chronic timepoint 38 days post-injury, WT^tg^ TBI mice showed persistent loss of thalamic neurons while MG IFNAR KO TBI mice were not significantly different from controls (Fig 8C). To investigate whether the subacute loss of thalamic neurons was correlated with the amount of TBI-induced cortical tissue loss, we measured cortical lesion area by tracing the lesion present in the overlaying cortex (Fig 8D). We found that cortical lesion area was not significantly different between WT^tg^ and MG IFNAR KO mice, confirming that both groups received brain injuries of similar severity (Fig 8E).

Furthermore, correlation analysis revealed that, in both WT^tg^ and MG IFNAR KO mice, there was no correlation between cortical lesion area and thalamic neuron density (Fig 8F). These findings support prior work demonstrating that thalamic neuronal loss is not a simple byproduct of cortical neuron loss and is influenced by secondary injury pathways such as activated immune cells, in this case, IFN-I activated microglia.

Finally, we utilized the Barnes Maze to evaluate if microglial IFNAR deficiency was sufficient to prevent TBI-induced deficits in spatial learning and memory. Barnes maze testing may be influenced by injury to several regions, including the hippocampus and thalamus. In our prior work, mice globally deficient in IFNAR had reduced memory impairment [12]. In the present study, we found that the main effects of time and condition (injury or genotype) were both significant during the four days of Barnes Maze training. There was no significant interaction between time and condition. To better understand how conditions differed from each other, we performed Tukey’s multiple comparisons test, which showed a significant increase in the latency to the escape between WT^tg^ TBI vs WT^tg^ controls on day 2 of training, and between MG IFNAR KO TBI vs MG IFNAR KO controls on day 4 of training (Fig 8G). On day 5 of testing, the probe trial showed that both WT^tg^ and MG IFNAR KO TBI groups had increased latency to the escape hole compared to controls (Fig 8I). Overall, this behavioral testing indicates that MG IFNAR KO failed to prevent TBI-induced spatial learning and memory deficits in the Barnes Maze.

## Discussion

Traumatic brain injury triggers a prolonged neuroimmune response that contributes to secondary neurodegeneration and worsens neurologic outcome. Neuroimmune modulation is a promising therapeutic strategy but must be carefully targeted given the contributions of the immune response to repair in addition to driving secondary neurotoxicity after TBI. Identifying cell-type–specific mechanisms that drive a reparative vs neurotoxic neuroimmune response is essential for development of targeted therapies. Here, we used microglial-specific IFNAR knockout mice to dissect the contribution of microglial type I interferon signaling on TBI pathophysiology. Our findings reveal that microglial IFNAR signaling selectively shapes microglial activation states, modulates key neuroimmune interactions, and disrupts neuronal integrity following TBI, specifically contributing to thalamic neuronal loss following TBI.

A central discovery of this study is that microglial IFNAR deficiency attenuates multiple facets of microglial reactivity—including reduced accumulation, decreased expression of ISGs, and diminished transcriptional activation—including very prominently in the thalamus. These molecular changes were accompanied by preservation of thalamic neurons at both acute and chronic time points. The clinical relevance of thalamic atrophy in TBI survivors has been increasingly recognized [37]. The convergence of regional transcriptomic, histologic, and cellular findings suggests that IFN-I–responsive microglia constitute a key mechanistic driver of thalamic neurodegeneration following TBI.

Our data also identify microglial IFNAR signaling as a regulator of phagocytic specificity. Although both WT^tg^ and MG IFNAR KO mice exhibited a TBI-induced increase in MBP ingestion, only WT^tg^ microglia showed robust phagocytosis of the presynaptic protein SNAP25. This selective effect aligns with prior studies demonstrating that type I interferons enhance microglial phagocytic programs and suggests that distinct phagocytic targets may rely on different upstream signaling pathways [38, 39]. Because excessive synaptic engulfment is increasingly recognized as a contributor to cognitive dysfunction across neurologic diseases, the reduction in SNAP25 phagocytosis in MG IFNAR KO mice highlights a potentially harmful IFN-I–dependent mechanism of synaptic injury after TBI [40–42]. Importantly, SNAP25 is part of the SNARE complex, a multi-protein complex that plays a key role in synaptic transmission and has been shown to be reduced in traumatic brain injury [43].

Beyond intrinsic microglial activation, microglial IFNAR signaling influenced crosstalk between the innate and adaptive immune systems. Microglial IFNAR deficiency reduced microglial and astrocytic *Cxcl10* expression across multiple brain regions, suggesting both autocrine and paracrine effects of microglial IFN-I signaling. Although total T cell numbers were not reduced, their penetration to subcortical structures was reduced, remaining instead in the cortex and extraparenchymal compartments. We also observed a reduction in microglial–T cell conjugates, supported by three independent assays, and a marked decrease in microglial MHC class I expression. Together, these findings indicate that microglial IFNAR signaling enhances antigen presentation capacity and promotes microglial–T cell engagement after TBI, providing a mechanistic pathway by which microglia may shape T cell localization and phenotype [32–34]. Further studies are needed to confirm an impact on T cell phenotype, and subsequently, on TBI pathophysiology.

Despite these significant effects on neuroimmune activation and thalamic neuroprotection, microglial IFNAR deficiency did not ameliorate TBI-induced deficits in spatial learning and memory. One reason for this divergence from the phenotype seen in global IFNAR-deficient mice is redundancy of IFN-I signaling across CNS cell types. Astrocytes retained substantial TBI-induced *Cxcl10* expression when microglial IFNAR signaling was blocked, highlighting the multicellular regulation of ISGs [14, 30]. These observations suggest that coordinated interferon blockade across multiple neural cell types may be required to achieve broad neuroprotection and behavioral rescue.

Finally, our single-cell data demonstrate that microglial IFNAR deficiency selectively reduces the proportion of interferon-responsive microglia but does not eliminate this subpopulation entirely. This incomplete suppression is consistent with the diverse upstream pathways capable of driving ISG expression, including type II interferons and IFN-independent nucleic acid sensing pathways [44]. The persistence of interferon-responsive microglia in MG IFNAR KO mice highlights the robustness of the ISG network and suggests that alternative or combinatorial strategies may be necessary to fully modulate this injury-associated microglial state.

In summary, our findings show that microglial IFNAR signaling contributes to TBI-induced neuropathology through its effects on microglial activation, synaptic engulfment, antigen presentation, and microglial–T cell interactions. While microglial IFNAR deficiency confers regional neuroprotection, the incomplete rescue of behavioral deficits underscores the importance of IFN-I signaling in other CNS cell types. These results highlight both the therapeutic potential and the limitations of cell-type–specific modulation of the interferon response and provide a framework for future efforts to target IFN-I signaling in TBI.

## Supplementary Material

1

Supplementary Files

This is a list of supplementary files associated with this preprint. Click to download.

- Supplementaltable1MicroglialsubclusterDEGs.xlsx
- Supplementaltable2nanostringDEGs.xlsx
- Supplementaltable3nanostringgeneontology.xlsx
- Supplementaltable4Statistics.xlsx

## Figures and Tables

**Table 1:** RNAscope probes and immunohistochemistry antibodies

RNAscope			
Gene target		Source	Identifier
*Irf7*		ACDbio	534541
*Spp1*		ACDbio	435191
*H2-K1*		ACDbio	1049831
*Slc1a3*		ACDbio	430781
*Hexb*		ACDbio	314231
*Cxcl10*		ACDbio	408921
*CD3e*		ACDbio	314721
*CD8a*		ACDbio	401681
Immunohistochemistry			
Antibody	Concentration	Source	Identifier
Iba1	1:200	FUJIFILM Wako	019–19741
NeuN	1:200	Abcam	177487

**Table 2:** Flow Antibodies

Antibody	Final Conc	Clone	Conjugate	Source	Identifier
CD16/32	1:100	2.4G2		BD Bioscience	553141
Cell sorting for single cell RNA sequencing					
CD45	1:150	30-F11	FITC	BD Bioscience	553080
Hoechst 33258	4ug/mL			ThermoFisher	H3569
**T cell panel**					
CD3	1:100	17A2	BV421	BD Bioscience	564008
CD44	1:100	IM7	BV510	BD Bioscience	563114
CD11a	1:100	2D7	FITC	BD Bioscience	553120
CD62L	1:100	MEL-14	PE	BD Bioscience	553151
CD8a	1:100	53–6.7	APC	BD Bioscience	553035
CD4	1:100	GK1.5	AF 700	Invitrogen	56-0041-82
Ly-6C	1:100	AL-21	BV421	BD Bioscience	562727
CD45	1:150	30-F11	FITC	BD Bioscience	553080
Ly-6G	1:100	1A8	PE	BD Bioscience	551461
LIVE/DEAD	1:100	NA	Fixable Blue	Invitrogen	L23105A
**MG-T cell flow panel**					
CD11b	1:100	M1/70	APC	BD Bioscience	553312
H2-Kd/H2-Dd	1:100	34-1-2S	AF488	BD Bioscience	567696
CD4	1:100	GK1.5	PE	BD Bioscience	553730
CD45	1:100	30-F11	PE-Texas Red	Invitrogen	MCD 4517
CD8a	1:100	53–6.7	BB700	BD Bioscience	566410
LIVE/DEAD	1:100	NA	Fixable Blue	Invitrogen	L23105A
MHC II (I-A/I-E)	1:100	M5/114.15.2	BV605	eBioscience	17-5321-82
TCRb	1:100	H57–597	APC	eBioscience	17-5961-82
CD11b	1:100	M1/70	BV786	eBioscience	417-0112-82
**Phagocytosis assay**					
MBP	1:400	P82H9	AF647	Biolegend	850910
Isotype control	1:400	MOPC-21	AF647	Biolegend	400155
SNAP25	1:400	SMI81	AF488	Biolegend	836313
Isotype control	1:400	MOPC-21	AF488	Biolegend	400133
CD45	1:200	30-F11	PE	BD Bioscience	553081
CD11b	1:200	M1/70	BV421	BD Bioscience	562605
LIVE/DEAD	1:100	NA	Fixable Blue	Invitrogen	L23105A

**Table 3:** Inhibitor cocktail stock solution

Inhibitor cocktail stock solution			
Reagent	Concentration	Manufacterer	Identifier
Cytochalasin D	2 mM	Tocris	1233
Wortmannin	2 mM	Tocris	1232
Pitstop 2	25 mM	Abcam	120687
Dynasore	40 mM	Tocris	2897
Bafilomycin A1	40 μM	Tocris	1334
Digestion mix			
Inhibitor cocktail	1:1000		
Collagenase IV	800 U/mL	Worthington	LS004189
DNAse-1	250 U/mL	Worthington	LK003172

## Data Availability

Raw data are available at GEO accessions: GSE328709.
