# Supplementary material for: Type I interferon signaling in microglia drives synaptic engulfment and neuronal loss following traumatic brain injury
Source: Res Sq. 2026 Jun 6:rs.3.rs-9785030. Preprint. [Version 1] doi: 10.21203/rs.3.rs-9785030/v1 (PMC13252574; doi:10.21203/rs.3.rs-9785030/v1)
Supplement: 1 [file NIHPPRS9785030V1-supplement-1.pdf]

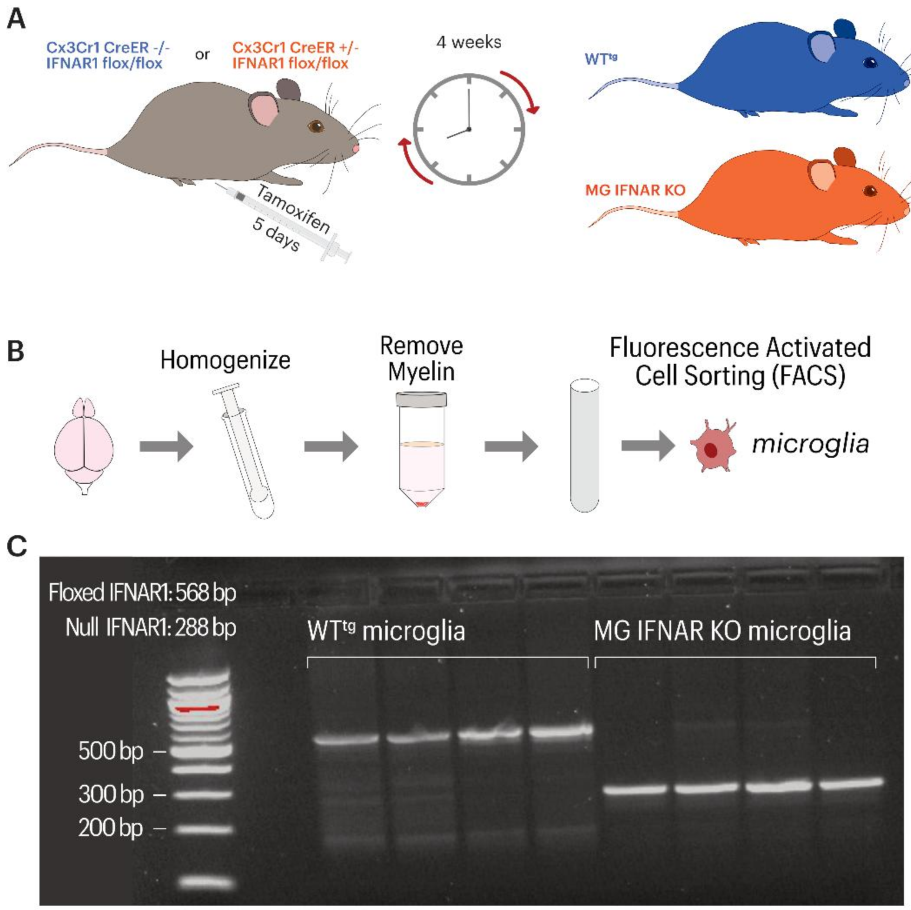


**Supplemental Figure 1. Generation of inducible, microglia-specific IFNAR knockout mice.** **(A)** Schematic of tamoxifen injection schedule. **(B)** Schematic of microglial cell isolation. **(C)**

Polymerase chain reaction showing confirmation of MG IFNAR KO in sorted microglia. n=4 $WT^{tg}$, n=4 MG IFNAR KO.

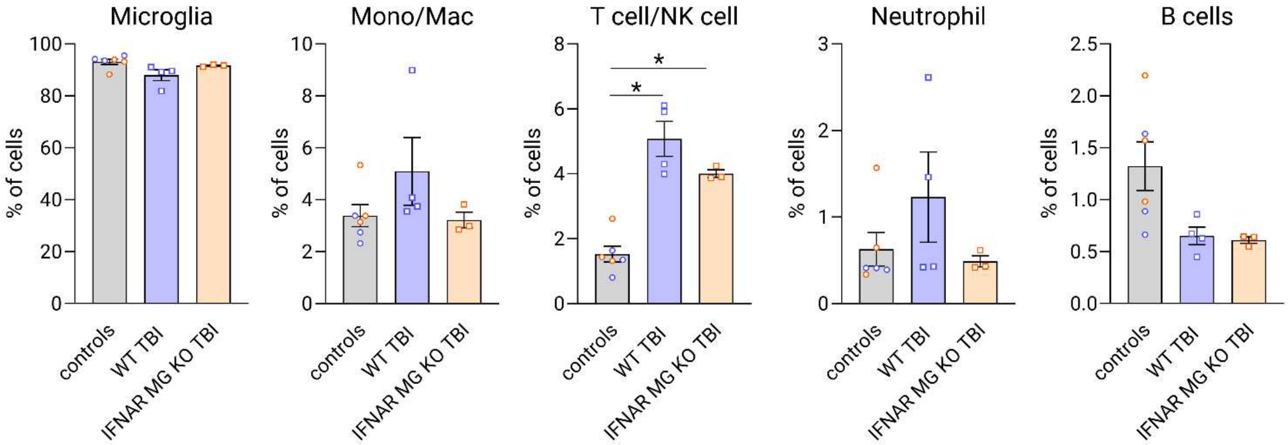


**Supplemental Figure 2**. **Proportion of immune cell types analyzed by single cell sequencing 7 days after TBI.** Bar graphs depict the proportion of each immune cell type yielded from CD45+ enriched single cell sequencing. Each dot represents a single subject; n=6 controls, n= 4 $WT^{tg}$ TBI, n=3 MG IFNAR KO TBI. One-way ANOVA with Sidak's multiple comparisons test.
